# Requirements of a cognitive-motor spatial orientation training for nursing home residents: an iterative feasibility study

**DOI:** 10.1007/s12662-021-00762-2

**Published:** 2021-10-14

**Authors:** Madeleine Fricke, Adele Kruse, Michael Schwenk, Carl-Philipp Jansen, Thomas Muehlbauer, Klaus Gramann, Bettina Wollesen

**Affiliations:** 1 Department of Biological Psychology and Neuroergonomics, TU Berlin, Fasanenstr. 1, 10623 Berlin, Germany; 2 Department of Human Movement Science, University of Hamburg, Hamburg, Germany; 3 Institute of Sports and Sports Sciences, Heidelberg University, Heidelberg, Germany; 4 Network Aging Research, Heidelberg University, Heidelberg, Germany; 5 Clinic for Geriatric Rehabilitation, Robert-Bosch-Krankenhaus Stuttgart, Stuttgart, Germany; 6 Division of Movement and Training Sciences/Biomechanics of Sport, University of Duisburg-Essen, Essen, Germany

**Keywords:** Long-term care, Spatial orientation abilities, Multicomponent training, Exercise, Aged

## Abstract

A sedentary lifestyle in nursing home residents is often accompanied with reduced life space mobility and in turn affects satisfaction with life. One of the reasons for this may be limited ability to find one’s way around the care facility and its environment. However, spatial orientation exercises might reduce these problems if they are integrated into an adequate cognitive-motor training. Therefore, we integrated six novel and target group-specific spatial orientation exercises into an established multicomponent cognitive-motor group training for nursing home residents and evaluated its feasibility. Forty nursing home residents (mean age: 87.3 ± 7 years) participated in the spatial orientation cognitive motor training (45–60 min, twice a week over a period of 12 weeks). The main outcomes included the feasibility criteria (adherence, completion time, acceptance, instructions, motor performance, materials/set up, complexity) and first measurements of mobility and satisfaction with life (SPPB [Short Physical Performance Battery], SWLS [Satisfaction with Life Scale]). Adherence increased over time. The increase was associated with the adaptions and modifications of the spatial orientation exercises that were made to meet the participants’ requirements. A positive trend was discerned for mobility and life satisfaction, comparing pre- and posttraining data. In summary, the feasibility analysis revealed that future interventions should consider that (a) instructions of demanding spatial tasks should be accompanied by an example task, (b) trainers should be encouraged to adjust task complexity and materials on an individual basis, (c) acceptance of the training should be promoted among nursing staff, and (d) surroundings with as little disturbance as possible should be selected for training.

## Introduction

Age-related physical and cognitive decline (Khan, Singer, & Vaughan, 2017) decreases mobility and quality of life (Colombo et al., 2017; Stöckel, Wunsch, & Hughes, 2017; Sverdrup et al., 2018). This promotes multimorbidity, which can lead to higher requirements for care and in turn to long-term care facility institutionalization (Akner, 2009). This relocation may cause some behavioral changes in terms of cognition and life space mobility. Many nursing home residents are sedentary, sitting or lying for longer periods during the day, even though they can walk independently without technical or human assistance (MacRae, Schnelle, Simmons, & Ouslander, 1996; Kuck, Pantke, & Flick, 2014). Jansen, Diegelmann, Schnabel, Wahl, and Hauer (2017) found that life space in nursing home residents is limited and a lot of time is spent in the private room. According to the concept of life-space mobility, life-space is defined as the social and physical environment in which the residents move on a daily basis. It is divided into four hierarchical areas (Tinetti & Ginter, 1990): (1) the residents’ private room, (2) the care section in which the private room is located, (3) all public areas within the facility, and (4) the area outside the facility. A larger life-space has been associated with a higher quality of life and good physical, cognitive, and psychosocial health (James, Boyle, Buchman, Barnes, & Bennett, 2011; Taylor, Buchan, & van der Veer, 2019). In contrast, the sedentary lifestyle of nursing home residents (Ice, 2002) promotes a reduction in independent activities of daily living (ADL; McGuire, Ford, & Ajani, 2006) and an increased risk of falling (Muir, Gopaul, & Odasso, 2012). The interaction of cognitive decline and decreasing mobility results in reduced physical activity and, in turn, in limited life-space mobility (Snih et al., 2012; Tung et al., 2014). This decreased mobility comes with less cognitive stimulation (Norton, Matthews, Barnes, Yaffe, & Brayne, 2014; Jansen et al., 2017), and eventually, a downward spiral in overall cognitive and physical function begins.

One of the highly complex cognitive abilities affected by age-related decline, even in mobile elderly, is spatial orientation and navigation (Taillade, N’Kaoua, & Sauzéon, 2016; Hilton, Muffato, Slattery, Miellet, & Wiener, 2020; Lokka & Çöltekin, 2020). Since finding a specific destination and the way back appears to be well-preserved in familiar environments (Rosenbaum, Winocur, Binns, & Moscovitch, 2012), finding one’s way in unfamiliar environments becomes more challenging in age (Kirasic, 1991; Wiener, Kmecova, & De Condappa, 2012; Muffato, Meneghetti, Doria, & De Beni, 2020). When moving into long-term care homes, residents get exposed to a spatially challenging new environment that might increase uncertainty about finding one’s way around the care facility and its environment. To orientate within and around the facility allocentric reference frames that allow coding of spatial information independent of the navigators’ current position and orientation are necessary (for example for taking an alternative route, if a previously learned route is blocked for cleaning). However, these functions decline with increasing age (Harris, Wiener, & Wolbers, 2012; Antonova et al., 2009; Gazova et al., 2013; Zhong & Moffat, 2018). Furthermore, an egocentric reference frame allows coding of spatial information concerning the navigator herself, like learning a route from your room to the training room by sensorimotor associations that couple egocentrically perceived landmark information (elevator, corner, etc.) with an egocentric action (turn left). Therefore, egocentric orientation is relevant to orientate in the nursing home environment, but remains preserved in older age (Moffat, Elkins, & Resnick, 2006; Colombo et al., 2017; Fricke & Bock, 2018).

For daily activities there are certain aspects within the interaction of the egocentric and allocentric reference frames according to life-space mobility: Both egocentric and allocentric reference frames can be used in combination for the entire life-space, but they might differ in their preferred use across life-space (Iaria, Palermo, Committeri, & Barton, 2009; Ruggiero, D’Errico, & Iachini, 2016). Everyday spatial activities use combinations of ego- and allocentric processes. For example, turning a corner leads to sensory feedback from the vestibular, proprioceptive and visual system that is processed within an egocentric frame of reference, but can be aligned with external visual information (landmarks visible behind the corner) in an allocentric frame of reference (Gramann, 2013).

The preferred use of one or the other spatial frames of reference might differ dependent on the available information and navigation goals. While in life-space 1, most or all objects and their locations are in sight (vista space) no mental representation is necessary to retrieve these locations and simple actions (e.g., turning around) can bring the desired object in sight. Beyond life-space 1, but inside the nursing home, the location of goals (e.g. the dining room) might not be directly visible and spatial representations are necessary to reach a desired goal location. The easiest is to follow a visual beacon that is the goal or that is located near the goal location. Routes can be recalled as a sequence of (egocentric) sensorimotor associations (turn left when leaving the room, then right at the elevator) or can be computed by combining the momentary location and orientation with respect to a survey representation of the goal location relative to other locations (e.g., one’s own room, the elevator, and the dining room). Survey knowledge can be used to flexibly compute different routes to known locations and even to locations that have not been visited before (Siegel & White, 1975). Using route representations becomes more demanding with an increasing number of routes to be remembered and an increasing number of associations within each remembered route. Allocentric representations (e.g., survey representation) allow for more flexible navigation strategies like taking shortcuts in case, for example, a route segment is blocked (Colombo et al., 2017). Outside the nursing home (life-space areas 3 and 4), route length and the number of sensorimotor associations increases, rendering it more difficult to remember the correct route. Here, landmarks can be used to orient and to compute the relative location of goals that are not directly in sight (triangulation). In this sense, the use of landmarks reduces the amount of sensorimotor associations to reach the bus station and allow for beacon navigation (go into the direction of a landmark) until further relevant information comes in sight.

In summary relocation to a nursing home might be a major challenge, as the residents have to adapt to the new environment. Interventions to enhance cognitive and motor fitness should address these aspects. However, there is evidence that even in old age brain and body plasticity remains (Erickson & Kramer, 2009; Moffat et al., 2006; Westlake & Culham, 2007). Therefore, implementing specific training interventions fostering mobility in nursing home residents is of major importance and can help to preserve cognitive and motor resources (Bischoff et al., 2021; Jansen et al., 2017; Demnitz et al., 2016; Sverdrup et al., 2018). Evaluations of exercise interventions indicate that especially cognitive-motor task training is beneficial to maintain or even improve cognitive and motor abilities in older age (Wollesen, Wildbredt, van Schooten, Lim, & Delbaere, 2020a; Wollesen & Voelcker-Rehage, 2014). Some studies investigating spatial orientation abilities demonstrated improvements in mental rotation (Whitlock et al., 2012), task-specific performance in virtual environments (Lövdén et al., 2012), and route finding (Kober et al., 2013). For example, Mitolo, Borella, Meneghetti, Carbone, and Pazzaglia (2017) implemented a spatial orientation training (route learning) for nursing home residents. As a result, they found significant improvements in route learning in post- and retention-testing after three months, compared to their control group doing cognitive activities (e.g., reading newspapers, watching movies with group debates). Beside this first framework of spatial orientation training, there is a lot of evidence that cognitive-motor training as a combination is more effective than the training of both aspects separately (Tait, Duckham, Milte, Main, & Daly, 2017). However, for mobility within a nursing home, the residents need both: the cognitive resources or abilities to find their ways through the environment and the physical capacity for daily movements into life spaces outside their room.

To overcome the sedentary lifestyle of nursing home residents and to enhance life-space mobility, spatial orientation training could be conducted with motor-fitness components for this target group. It can be assumed that three pathways of physical training are beneficial for this target group: (1) physical activity and target-group specific exercise to increase cognitive and motor fitness, (2) social involvement with related psychosocial parameters and mental health, and (3) enhanced cognitive stimulation, also associated with cognition and physical and global functioning promoting independence in everyday life and quality of life (Kramer & Colcombe, 2018; Newsom, Shaw, August, & Strath, 2018; Smith et al., 2019; Warbuton & Bredin, 2017).

Moreover, to design innovative and effective spatial orientation training, existing recommendations for cognitive-motor interventions can be applied: the training has to be implemented for a minimum of 3 months and should be performed at least 60 min per week (Wollesen, et al., 2020a; Herold, Müller, Gronwald, & Müller, 2019). The training should be adapted to the participants’ individual preconditions (e.g., movement limitations, hearing impairments, low vision) (Herold et al., 2019), and challenge participants progressively according to the FITT principle (Frequency, Intensity, Time, Type; Garber et al., 2011). Additionally, the spatial orientation exercises should target the use of both spatial reference frames, i.e., the combination of egocentric and allocentric tasks should train the well-preserved use of egocentric reference frames in combination with the (less well-preserved) allocentric reference frames to improve overall spatial orientation abilities.

To our knowledge, no study implemented a training combining cognitive-motor exercises and spatial orientation exercises in a nursing home setting, yet. According to Lancaster and Thabane (2019), new interventions should be developed following a theoretical model and then tried out in a smaller group of participants and then adopted and modified if necessary. This process needs to be conducted with a variety of qualitative measurements and observations. The overall aim of this study was to integrate novel spatial orientation exercises from different target groups into an established cognitive-motor group training for nursing home residents based upon the current literature.

1. Incorporating evidence-based spatial orientation exercises from different target groups.
2. Adapting exercises from different target groups to the requirements of nursing-home residents.
3. Integration of the novel spatial orientation exercises into an established cognitive-motor group training.
4. Testing of the feasibility of the spatial orientation cognitive-motor training for nursing home residents, based upon the following feasibility criteria: Adherence, completion time, acceptance, instructions, motor performance, materials/ set up, complexity (El-Kotob & Giangregorio, 2018).
5. Analyzing effects on mobility and life satisfaction on nursing home residents.

We hypothesize that our novel spatial orientation cognitive-motor training is feasible for nursing home residents and will prolong decline or improve mobility and satisfaction with life.

## Methods

This study determined the feasibility of innovative spatial orientation exercises for nursing home residents. The implementation and description of this feasibility study were based on the guidelines of the extended CONSORT statement (Eldridge et al., 2016).

### Study design and participants

The study was planned as a controlled trial including pre-and post-testing. A total number of *N* = 40 nursing home residents (87.3 ± 7 years; Table 1) were recruited in three nursing homes in northern Germany. They received the opportunity to participate in a maximum of 24 group training sessions (twice per week, each 45–60 min). The participants were recruited through a pre-selection of the nursing staff and occupational therapists, following the inclusion criteria: ability to (1) participate in group activities, (2) understand and (3) execute simple instructions including visual implementations of landmarks. No exclusion criteria were applied. Data collection and interventions were conducted from September 2019 to December 2020. The integration of the control group was not possible due to coronavirus disease 2019 (COVID-19)-related restrictions.

**Table 1:** Baseline characteristics of participants and dropouts

	*n*	NH‑A (*n* = 6)	*n*	NH‑B (*n* = 13)	*n*	NH‑C (*n* = 7)	*F* value	*p *value and $\eta _{p}^{2}$	*n*	Dropouts (*n* = 14)	*F* value	*p *value and $\eta _{p}^{2}$
Age (mean ± SD)	6	87.3 ± 6.1	12	87.8 ± 4.6	7	86.3 ± 5.5	0.15	0.86 0.01	12	87.4 ± 9.6	0.01	0.94 0.00
BMI (mean ± SD)	6	30.5 ± 5.4	13	25.6 ± 5.4	7	27.5 ± 3.8	1.72	0.20 0.01	5	26.8 ± 2.9	2.27	0.14 0.07
MMSE (mean ± SD)	6	22.5 ± 3.2	–	–	7	26.6 ± 2.1	6.46	0.03* 0.37	13	21.5 ± 8.0	1.58	0.22 0.06
SF-12 Mental Health (mean ± SD)	6	58.4 ± 4.2	–	–	7	54.6 ± 7.7	1.00	0.34 0.08	12	51.7 ± 9.9	1.78	0.20 0.07
SF-12 Physical Health (mean ± SD)	6	43.6 ± 10.1	–	–	7	29.1 ± 6.7	8.14	0.02* 0.43	12	42.8 ± 6.2	3.45	0.08 0.13
Handgrip strength right (kg) (mean ± SD)	6	15.2 ± 2.2	–	–	7	18.4 ± 7.6	0.87	0.37 0.07	12	19.3 ± 5.0	1.08	0.31 0.05
Handgrip strength left (kg) (mean ± SD)	6	17.0 ± 3.8	–	–	7	17.9 ± 4.8	0.11	0.75 0.01	12	19.7 ± 5.1	1.26	0.27 0.05
SWLS (mean ± SD)	6	25.0 ± 7.4	13	25.4 ± 3.2	7	26.9 ± 4.2	0.27	0.77 0.02	13	24.8 ± 5.5	0.23	0.63 0.01
Females, *N* (%) Males, *N* (%)	–	5 (80.3) 1 (19.7)	–	13 (100.0) 0 (0.0)	–	6 (85.7) 1 (14.3)	–	–	–	12 (85.7) 2 (14.3)	–	–

*NH‑A* nursing home A, *NH-*B nursing home B, *NH‑C* nursing home C, *SD* standard deviation, *BMI* Body Mass Index, *MMSE* Mini Mental State Examination, *SF-12* Short Form 12, *SWLS* Satisfaction with Life Scale, *SPPB* Short Physical Performance Battery

*p-value is less than 0.05 and statistically significant

The study was conducted in accordance with the latest version of the Declaration of Helsinki (2013). Written informed consent was obtained prior study enrollment from all participants or their legal guardians. This trial was granted permission by the local ethics committee faculty of psychology and human movement science, University of Hamburg (No. 2019_248 and 2020_310), and was retrospectively registered at DRKS.de with registration number DRKS00020518 on February 19, 2020. It is part of the PROfit project (for study protocol, cf. Wollesen et al., 2020b).

### Development of spatial orientation exercises

The development of spatial orientation exercises for the target group of nursing home residents followed five steps:

1. Incorporating evidence-based spatial orientation exercises from different target groups

The identified exercises of previous research (Mitolo et al., 2017; Lövdén et al., 2012; Kober et al., 2013) addressed the egocentric reference frame as well as the allocentric one, encouraging abilities like route knowledge, survey knowledge, map use, or direction pointing.

2. Adapting exercises from different target groups to the requirements of nursing-home residents

Six spatial orientation exercises—(1) “Direction twist”, (2) “Parachute pointing”, (3) “Known or unknown?”, (4) “Mental exploration journey”, (5) “Floor plan bingo” and (6) “Where is mine?”—were created in a balanced proportion of egocentric and allocentric demands (Table 2). To create the six everyday-life-relevant spatial training components for the nursing home residents, individual photos of salient objects (so-called landmarks) in and around the facilities were taken and simplified floor plans were designed for the training. The practicability of these tasks was piloted within a digital intervention with independently living older women (*N* = 6). The piloting results were used to modify the tasks according to the requirements of the more vulnerable target group of nursing home residents. For example, landmarks and floor plans were printed in high contrast and large formats to prevent potential difficulties due to the visual impairments frequently occurring in nursing home residents (Bischoff et al., 2020). Finally, the exercises were adapted target-group-specifically in terms of frequency, intensity, time, and type (FITT principles, Garber et al., 2011). Adaptions were made according to the observation protocols and the individuals’ performance.

3. Integration of the novel spatial orientation exercises into an established cognitive-motor group training

**Table 2:** Description of the spatial orientation exercises

Exercise name	Addressed spatial reference frame *planned duration*	Material	Description	Initial implementation *session number of implementations*	Progression *session number of implementations*
(1) Direction twist	Egocentric *5* *min*	1 list of landmarks	Participants are standing behind a chair (grabbing it with one hand) or sitting. The trainer names a landmark/everyday object and the participants are supposed to point in the direction of it with their hands or their feet. Different foot positions are announced (parallel stand, semi-tandem stand, tandem stand)	Landmarks, which depict memorable locations/things within one’s room, the care section and the facility (life space area 1–3) *Training session 1 & 2*	Landmarks, which depict memorable locations/things in and around the facility (life space area 3–4) *Training session 13 & 14*
(2) Parachute pointing	Egocentric & allocentric *5* *min*	1 play parachute (diameter 3 meter) 1 Redondo ball 1 list of landmarks	Participants are sitting in a circle, each grabbing two handles of the play parachute, a ball circles and jumps on the play parachute. The direction in which the ball is moved points to landmarks in the surroundings as if on a 360° scale. The trainer names a landmark and the participants are supposed to (1) move the ball into the color field/direction in which the landmark is located and (2) move the play parachute up and down according to the height of the landmark (in floors)	Landmarks, which depict memorable locations/things within the care section and the facility (life space area 1–3) *Training session 3 & 4*	Landmarks, which depict memorable locations/things in and around the facility (life space area 3–4) *Training session 15 & 16*
(3) Known or unknown?	Allocentric *10* *min*	12 photos depicting potentially known landmarks 6 photos depicting unknown landmarks 1 massage ball per participant	Participants are standing, walking in place or sitting. The trainer shows one landmark photo at a time and the participants have to decide whether it belongs to their environment or not. (1) For the first photo set (landmark 1–9) the participants circle the ball around their heads if a known place is depicted. If not, they pass the ball from hand to hand over their heads. (2) With the second photo set (landmark 10–18), other movements are carried out. If the photo depicts a known place, the participants throw the ball with their left hand, if not, with their right hand	Photoset depicting landmarks within the care section and the facility (life space area 2–3) *Training session 5 & 6*	Photoset depicting landmarks in and around the facility (life space area 3–4) *Training session 17 & 18*
(4) Mental exploration journey	Egocentric *5* *min*	1 movement journey (written version)	The trainer narrates a journey and adds movements to crossed landmarks. Participants imitate the movement simultaneously. The journey proceeds twice. If there is time left, the same journey is retold backward	Movement journey narrating an activity from one’s room to the grounds of the facility (life space area 1–3). *Training session 7 & 8*	Movement journey narrating an activity from one’s room to an event in the surrounding area of the facility (life space area 1–4). *Training session 19 & 20*
(5) Floor plan bingo	Allocentric *5* *min*	1 floor plan per participant 1 floor plan with labeled landmarks (trainers’ plan for verification) 1 list of landmarks	Participants are standing or sitting and perform one movement continuously (e.g., walking on the spot or tapping with the tips of their feet) while holding a floor plan in their hands. The trainer names a landmark and the participants are supposed to find its location on the plan. When they find the landmark on the plan, the participants shout “Bingo!” and the trainer gives feedback. If the position was correct, the participant is allowed to pause until the next landmark is named	Floor plans showing the care section and the facility (life space area 2–3). *Training session 9 & 10*	Floor plans showing the facility and maps of the surrounding area of the facility (life space area 1–4). *Training session 21 & 22*
(6) Where is mine?	Allocentric *10* *min*	1 list of everyday objects	Participants are standing or sitting. The trainer associates four rooms/locations with different movements, the participants repeat them. Afterward, the trainer names an everyday object and the participants perform the movement that belongs to the room, where the object is located	Implemented rooms/locations within the own room, the care section and the facility (life space area 1–3) *Training session 11 & 12*	Implemented rooms/locations within the care section, in and outside the facility (life space area 2–4) *Training session 23 & 24*

To comply with the recommendations for an efficient training program (Herold et al., 2019), 24 training sessions were planned, each consisting of five parts (warm-up and mobilization; balance, coordination and cognitive exercises; aerobic exercises; strength exercises; cool down; for further details cf. Cordes et al., 2019; Bischoff et al., 2020). Only one novel spatial orientation exercise was integrated per session, mainly into the cognitive exercise of the original training. It was repeated in the following training sessions (initial implementations). The two sessions conducting the progression of the spatial orientation exercises were scheduled 6 weeks later (Table 2). The training sessions took place twice a week and lasted for 45–60 min.

4. Testing of the feasibility of the spatial orientation cognitive-motor training for nursing home residents

The spatial orientation cognitive-motor training was implemented and iteratively modified in terms of completion time, instructions, complexity, materials and set up in three nursing homes.

5. Analyzing effects on mobility and life satisfaction on nursing home residents

### Baseline measurements

To verify comparability per group of participants from the three care facilities, baseline measurements of cognitive status, quality of life, and muscle strength were conducted.

#### Mini Mental State Examination

The German version of the *Mini Mental State Examination *(MMSE, Folstein, Robins, & Helzer, 1983) is a widely used 10 min tool to detect cognitive impairments in older adults with a good test–retest reliability (r = 0.80–0.95; Baek, Kim, Park, & Kim, 2016). Concerning validity of the MMSE, the cross-correlation with the Wechsler Adult Intelligence Scale score revealed a correlation coefficient of r = 0.78 (Folstein, Folstein, & McHugh, 1975). The summed value of the individual items indicates the participant’s current cognitive status. The items of the MMSE consist of tasks addressing orientation, registration, memory, calculation and attention, naming, repetition, comprehension, reading, writing, and drawing. The maximum score is 30 points (Cockrell & Folstein, 2002).

#### 12-Item Short-Form Health Survey

All participants completed a standardized short form of the SF 36, the 12-Item Short-Form Health Survey (SF-12; Bullinger, Kirchberger, & Ware, 1995). It is a widely used and reliable measurement to assess health-related quality of life (Cronbach’s alpha (α) ranged between 0.57 and 0.94; Bullinger & Kirchberger, 1998). Analyses included a norm-based scoring algorithm that resulted in two scores, with higher scores indicating better physical and mental health (Ware, Kosinski, & Keller, 1995).

#### Hand Grip Strength

In our study, *Hand Grip Strength* was registered using the established JAMAR hydraulic hand dynamometer (Mathiowetz, 2002). Decreased handgrip strength has been shown to be an indicator of frailty (Rantanen et al., 1999; Bohannon, 2008), muscle strength, mortality, quality of life, and/or cardiac health (Norman, Stobäus, Gonzalez, Schulzke, & Pirlich, 2011; Wollesen & Voelcker-Rehage, 2019). Hand Grip Strength is a reliable tool for use in clinical practice (ICC values 0.85–0.98) and healthy populations (Peolsson, Hedlund, & Öberg, 2001). Participants were asked to execute two alternating trials per hand. The highest value gained for each hand was integrated into the analyses.

### Feasibility of spatial orientation training tasks

To assess the feasibility of the novel spatial orientation cognitive-motor training for nursing home residents, the trainers registered attendance, reasons for absence, and dropouts (according to El-Kotob & Giangregorio, 2018). In nursing homes B and C, standardized observation protocols were monitored by an independent evaluator or by the trainers, depending on how many external persons were allowed to enter the care facility due to COVID-19. These protocols were developed based on the experience gained from preliminary tests with independently living elderly and individual nursing home residents as well as first experiences in real conditions in nursing home A.

#### Adherence

To reflect adherence, an attendance rate (mean of attending participants per session and per facility) and a retention rate (residents participating until the end of the program/all participants that started the training) were calculated.

#### Completion time of the training tasks

The novel spatial orientation tasks were tested in prior work by the future assessors with independently living older adults and nursing home residents. Therefore, a completion time between 5 and 10 min was derived for each novel spatial orientation task for the nursing home residents. During the interventions, the actual execution length was documented individually for each exercise. A mean value was calculated for the completion time of all initial implementations and progressions.

#### Qualitative content analysis of the training tasks

Additionally, structured observation of the execution of spatial orientation exercises was conducted using standardized protocols. These were subsequently evaluated using qualitative content analysis according to Mayring (2014). Each comment found in the observation protocols was assigned to a category (e.g., “acceptance” or “instructions”). The categories were not predefined but defined by two raters according to the content of the observation.

### Outcome measures

To gain first data of motor, cognitive and emotional benefits, the feasibility assessment was accompanied before and after the intervention by measurements addressing physical functioning (Short Physical Performance Battery), psychosocial wellbeing (Satisfaction with Life Scale), and structured standardized observations of the training sessions.

#### Satisfaction with Life Scale

A global determination of life satisfaction was measured with the *Satisfaction with Life Scale* (SWLS, Diener, Emmons, Larsen, & Griffin, 1985). The German translation (Janke & Glöckner-Rist, 2012) includes five items that are rated by a seven-point Likert scale, “1” standing for “strongly disagree” and “4” indicating “strongly agree”. Higher scores represent a higher level of life satisfaction (Glaesmer, Grande, Braehler, & Roth, 2011). Convergent validity was shown for example correlating the SWLS with the single-item global life satisfaction measure (r = 0.56, van Beuningen, 2012). The internal reliability of the SWLS is moderate (Cronbach’s α = 0.78; Vasser, 2008).

#### Short Physical Performance Battery

The *Short Physical Performance Battery* (SPPB) is a standardized and valid measure for lower extremity functionality (Guralnik et al., 1994) with a high test–retest reliability (0.87, 95% confidence interval [CI] 0.77–0.96; Gómez, Curcio, Alvarado, Zunzunegui, & Guralnik, 2000). It consists of three tests including balance, leg strength, and gait speed that are combined to calculate a score (0–12 points). For balance testing, three positions must be maintained for a maximum of 10 s (side-by-side, semi-tandem, tandem stands). Leg strength is calculated by five chair-rises at maximum speed. Gait speed was measured using a stopwatch that was started when participants began walking at their usual everyday speed and stopped when they passed a cone at a distance of 4 m. The SPPB is used in different populations (e.g., older age, older hospitalized patients, nursing home residents) and is associated with mobility, lower extremity performance and fall risk (Lauretani et al., 2018; Volpato et al., 2010).

#### Performance in spatial orientation exercises

Participants’ performance was rated by the independent evaluators or the trainers, using a four-point Likert scale for each spatial orientation exercise, “1” indicating that the exercise was not performed and “4” representing a performance without hesitation. This scale was developed based on the experience with observation protocols gained from preliminary testing independently living elderly and individual nursing home residents. The mean values for each of the six exercises were calculated as a performance score. In the analysis, the mean values of initial implementations of the exercises and their progression were compared statistically using Wilcoxon tests (α < 0.05).

## Results

**Fig. 1:**
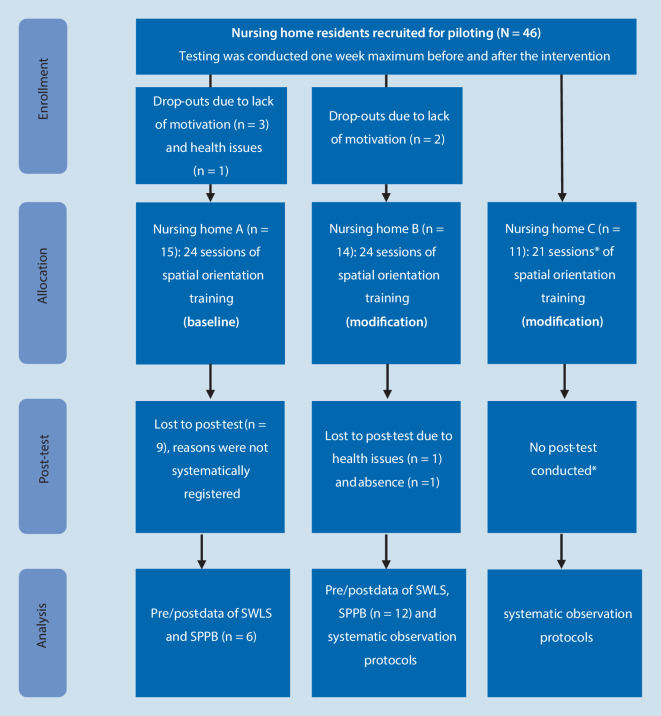
Flowchart CONSORT statement. *Asterisk* due to COVID-19-(coronavirus disease 2019)-related restrictions. *SWLS* Satisfaction with Life Scale, *SPPB* Short Physical Performance Battery

Forty nursing home residents participated in the spatial orientation training (Fig. 1). There were significant differences in the baseline characteristics between participants of nursing home A (NH-A) and nursing home C (NH-C). Participants in NH‑A scored significantly lower on the MMSE and higher on the Physical Health Scale of the SF-12, compared to nursing home C (NH-C) (Table 1).

First, the residents of NH‑A were assigned into two groups (intervention group: spatial orientation cognitive-motor training; active control group: basic cognitive-motor training (Bischoff et al., 2020) by stratified randomization (sex, age, MMSE)). High dropout quotes led to a merged group receiving the spatial orientation training after the tenth session. Due to COVID-19-related restrictions, the study design had to be changed for NH‑B and NH‑C. In both nursing homes, smaller training groups needed to be implemented without a matching active control group, and stratification was conducted per care section. In NH‑C the training had to be paused for one and a half weeks and continued afterward in small groups (2–3 participants). One resident subsequently dropped out of the training. Post-tests could not be conducted (Fig. 1). No adverse events were reported during or related to the assessments and training of this study.

### Feasibility

#### Adherence

The spatial orientation training was conducted in three nursing facilities consecutively, after the first implementation in NH‑A modifications were made, which improved adherence in NH‑B and NH‑C. In NH‑A, 15 nursing home residents started the spatial orientation training. On average, 36.9% (*n* = 5.5) of these participants attended the training sessions, 40% (*n* = 6) attended until the end of the program (9 dropouts). The highest adherence was documented in NH‑B. All but one of the 14 participants regularly attended the training sessions (attendance rate: 94.6%, retention rate: 92.9%, 1 dropout). In contrast to the other facilities, participants in NH‑B were very consistently reminded of their training and accompanied on their way, if necessary. In NH‑C, on average 68.6% (*n* = 7.6) of the 11 enrolled participants attended the training sessions, and 63.6% (*n* = 7) of them participated until the end of the program (4 dropouts).

In NH‑A the baseline spatial orientation training was conducted and iteratively adapted for NH‑B and NH‑C. Reasons for absence and dropout were not systematically registered in NH‑A. In NH‑B and NH‑C, participants missed training sessions due to health issues (58.8%), low motivation (5.9%), competing activities like birthdays (8.8%), and for 26.5% the reasons for absence were not mentioned. Twenty percent of the attending participants in NH‑B and NH‑C left the spatial orientation training before the program ended, mainly because of health issues (60%) and loss of motivation (40%). After a few weeks, two nursing home residents joined the training spontaneously in NH‑A. They were not considered in measurements and data analysis.

#### Completion time of the training tasks

Four of the six spatial orientation exercises were designed to take 5 min each, the other two took 10 min each (Table 2). Averagely, the completion time of all spatial orientation exercises was higher than expected (mean + 1.3 min; standard deviation [SD] ± 3.3 min), which was more apparent for the initial implementations of the novel exercises than for the progression.

The planned completion times of the exercise tasks “Mental exploration journey” (5 min) and “Where is mine?” (10 min) were most feasible. Their duration deviated averagely less than one minute from the intended time (mean + 0.7 min, SD ± 1.2 min; mean −0.5 min, SD ± 1.2 min). “Known or unknown?” did not take as long as expected in initial implementation (mean −2.4 min, SD ± 2.6 min), but was in line with the planned completion time in progression. On average, “Direction twist” took 2.5 min longer than planned in the implementation, and 3.5 min in the progression (mean +3 min, SD ± 1.6 min). “Parachute pointing” and “Floor plan bingo” required almost twice as much time in implementation as planned (mean +4.6 min, SD ± 5.5 min; mean +4.6 min, SD ± 1.4 min). The completion time of “Parachute pointing” decreased considerably and reached a feasible level in progression (mean +0.7 min, SD ± 1.2 min) but for “Floor plan bingo” additional time was still necessary (mean +3.8 min, SD ± 1.2 min).

#### Qualitative content analysis of the training tasks

The content analysis integrated *n* = 45 standardized observation protocols of different training sessions. It resulted in five categories: acceptance, instructions, motor performance, material/set up, and complexity. Since the comments on “complexity” showed a content-related pattern, we created the following subcategories: low complexity, adequate complexity, and high complexity. Two reviewers independently screened and assigned the comments to the categories (acceptance, instructions, motor performance, material/set up, and complexity). Disagreements were resolved by discussions.

#### Acceptance

Comments were assigned to the category “Acceptance” if they indicated an engagement or emotion regarding the exercise. In total, 57 comments on acceptance were found in the protocols, many of them expressing a positive mood (e.g., “*participants had fun*”, “*everyone joins in*”, “*very popular among participants*”). In contrast, observations of resentment (e.g., “*participant does not like the exercise*”, “*participant is disappointed that she can’t find the landmarks on the floor plan*”) were registered especially for the “Floor plan bingo” exercise. This exercise differs conceptually from the others as it requires locating specific landmarks as accurately as possible from a bird’s eye view.

#### Instructions

Twenty-four comments were identified for the category “Instructions”, pointing out both methodological and contextual difficulties. For example, some exercises were explained too quickly or the transition from landmark to landmark within the exercise was too fast to allow the participants to perform the exercise (e.g., “*tasks are too quick in a row*”). Participants had difficulties in understanding the instruction if multiple and individually correct solutions were mentioned. In “Floor plan bingo” and “Where is mine?”, several and repeated explanations were necessary. In some cases, the trainers reacted to such situations by splitting the exercise and their instruction into several components, which were first carried out separately and subsequently combined. As a consequence, simplified, potentially more comprehensible instructions were created for both tasks subsequently.

#### Motor performance

The category “Motor performance” represents the physical performance during the spatial orientation exercises. The 41 comments show that the motor demands in “Direction twist” led to executions at different balance levels (e.g., “*four participants turn their feet in the correct direction, but are not able to stand up*”, “*two participants perform the task in the tandem stand, one attempts to*”). However, some comments suggested that the motor demands masked the spatial orientation task in “Parachute playing” and “Direction twist” (e.g., “*ball seems to be hard to control for indicating a direction on the play parachute”, “pointing with the hands is easier, two participants skip the tasks for the feet*”). For these exercises, less demanding movement variations were added afterwards.

#### Material/set up

Twenty-eight comments were assigned to the category “material/set up”. It was observed that environmental conditions sometimes affected the training (e.g., “*It is very noisy in the facility. Participants felt it hard to concentrate*.”). As space is limited in many facilities, often no separate room for group therapy and exercise programs is available. Therefore, activities are necessarily conducted in the dining rooms or within the care sections, where everyday life goes on at the same time. This generally complicates the implementation of activities and reduces their quality. Hence, separate rooms for group activities would be desirable as a standard for newly built facilities.

The copies of the landmarks and floor plans were visually challenging for the participants due to their size (e.g., “*participants ask for larger photos*”, “*participants have visual difficulties in reading the floor plan*”). However, participants forgot to bring their visual aids to the training and had to get them, which repeatedly interfered with the timing of the training sessions. We therefore recommend at least a size of 15 × 20 cm for photos, and for the floor plans a minimum size of 30 × 42 cm.

In general, the observation protocols showed that if inappropriate material complicated the execution, the trainer indicated a solution autonomously (e.g., “*ball rolls away inconveniently when falling, cushions used instead*”). These adjustments were adopted for future implementations.

#### Complexity

In total, 102 comments identified how challenging the spatial orientation exercises were for the nursing home residents. They were divided into the subcategories “low complexity” (6 comments), “adequate complexity” (50 observations), and “high complexity” (46 comments).

In NH‑B, there were no comments that rated the spatial orientation exercises as simple to perform. However, there are references to a low complexity in NH‑C for the “Parachute pointing” and the progression of “Direction twist” (e.g., “*easy to do for all participants*”). For two of the spatial orientation exercises (“Where is mine?”, “Known or unknown”), intuitive performance was repeatedly observed, both in the initial implementation and in the progression (e.g., “*Participants complete the task without any hesitation*.”). The same pattern is found for “Parachute pointing” and “Mental exploration journey”, but only in NH‑C. An adequate complexity was first observed for “Floor plan bingo” and “Direction twist” in the progression (e.g., “*increasingly faster response*”*, *“*realization of the exercise improved over time*”).

Observations in “Known or unknown” suggest that participants struggled to remember the move, they were asked to perform, depending on whether or not they knew the location of the photo. For “Parachute pointing”, comments indicated a high degree of difficulty in the progression of the exercise in NH‑C, apparently depending on the distance of the landmarks to the facility (e.g., “*Performance becomes more uncertain the further away from the house it is*”).

Some of the participants living in NH‑C were challenged by the progression of the “Mental exploration journey”, especially when they were supposed to retell the journey and the added movements backward. “Direction Twist” seemed to be challenging for the participants in the implementation (e.g., “*need time to understand what to do*”); however, this was not reported for the progression.

The most frequent comments on a high level of demand were found for “Where is mine” and “Floor plan bingo”, both in the initial implementation and in the progression (e.g., “*exercise is difficult and was canceled*”, “*participant copies solution from another participant, finds exercise too difficult*”).

### Outcome measures

#### Satisfaction with Life Scale and Short Physical Performance Battery

The scores of the SWLS increased in NH‑A and NH‑B. For the SPPB, a different result was obtained: the participants in NH‑A showed a decrease, while SPPB scores increased in NH‑B after 12 weeks of training. Data for NH‑C are missing due to COVID-19-related restrictions. Statistical comparisons of pre- and post-training data showed no significance (Table 3).

**Table 3:** Short Physical Performance Battery (SPPB) scores and Satisfaction with Life Scale (SWLS) scores before and after 12 weeks of training

	*n*	Baseline NH‑A (mean ± SD)	Post-training NH‑A (mean ± SD)	Wilcoxon Test (*z-*value*; p‑*value)	*n*	Modification 1 NH‑B (mean ± SD)	Post-training NH‑B (mean ± SD)	Wilcoxon Test (*z-*value*; p‑*value)
SPPB	6	4.3 ± 2.2	4.2 ± 2.4	−0.11; 0.92	12	3.8 ± 2.7	5.4 ± 2.8	−0.67; 0.51
SWLS	6	25.0 ± 7.4	25.5 ± 5.3	−0.18; 0.85	12	25.3 ± 3.3	26.3 ± 7.4	−1.73; 0.08

*NH‑A* nursing home A, *NH-*B nursing home B, *SD* standard deviation, *SPPB* Short Physical Performance Battery, *SWLS* Satisfaction with Life Scale

#### Performance in spatial orientation exercises

**Fig. 2:**
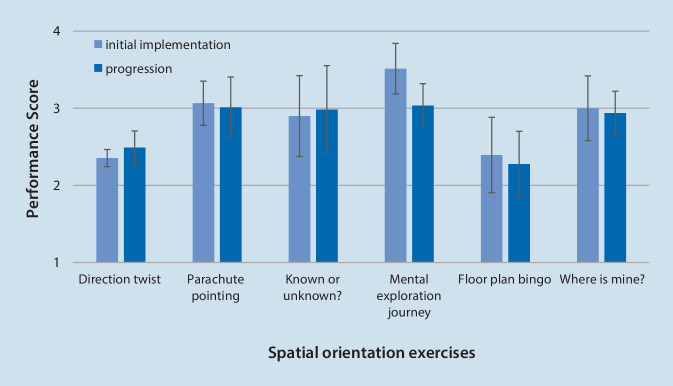
Descriptive (mean ± standard deviation) analysis of participants’ performance in spatial orientation exercises. (“1” = exercise not performed; “2” = exercise performed with hesitation; “3” = exercise performed with light hesitation; “4” = exercise performed; Wilcoxon tests did not show statistical changes between initial implementation and progression of the spatial orientation exercises)

As shown in Fig. 2, the spatial orientation exercises were performed with varying degrees of hesitation in initial implementation (2.9 ± 0.5) and progression (2.8 ± 0.5). While the “Floor plan bingo” and the “Direction twist” were the most challenging to perform, this improved in the progression at least for the “Direction twist”. The other four exercises reached a value in the initial implementation, which reflects a slightly hesitant start into the exercises. Wilcoxon tests were used to compare the performance changes between initial implementation and progression. These changes did not reach any significance (“Direction twist”: z = −0.82, *p* = 0.41; “Parachute Pointing”: z = −0.45, *p* = 0.65; “Known or unknown”: z = −1.6 *p* = 0.11; “Mental exploration”: z = −1.6, *p* = 0.11; “Floor plan bingo”: z = −1.07, *p* = 0.28; “Where is mine”: z = 0, *p* = 1.0).

## Discussion

This study aimed to integrate novel spatial orientation exercises into an established cognitive-motor group training and to evaluate its feasibility for nursing home residents. Therefore, 40 nursing home residents participated in a novel spatial orientation cognitive-motor intervention. Feasibility was identified by analysis of adherence, completion time, and structured training observations (addressing acceptance, instructions, motor performance, materials/set up, and complexity of the spatial orientation exercises). Besides this, the first preliminary effects of the spatial orientation cognitive-motor training on mobility (SPPB) and life satisfaction (SWLS) were assessed for nursing home residents.

### Feasibility

Regarding the results of feasibility, the structured qualitative content analysis suggests the feasibility of the spatial orientation exercises at many points but also reveals practical difficulties (e.g., tasks difficult to understand, too demanding, unsuitable materials) leading to indications for necessary modifications in future implementations. This was particularly evident for two of the exercises, “Floor plan bingo” and “Direction twist”, which were also rated as most challenging. The explanation for these difficulties is two-fold: the “Direction twist” task requires movement of the feet, which might be challenging for this cohort with low scores in SPPB that indicate reduced lower extremity performance. For the “Floor plan bingo”, we suggest that the allocentric nature of the task led to higher performance difficulties. As abilities concerning the allocentric reference frame are more likely to be affected by an age-related decline (Harris et al., 2012; Antonova et al., 2009; Gazova et al., 2013; Zhong & Moffat, 2018), the allocentric orientation exercises could have been more demanding for the participants than the egocentric ones. Nevertheless, to enhance the feasibility of these specific tasks, the instructions, complexity, and materials need to be modified (e.g., instructions followed by an example task, selection of more salient landmarks). As stated above, both spatial frames of reference are often combined but with increasing age, egocentric strategies become prevalent. The intervention trains not only allocentric strategies but also the integration of egocentric with allocentric spatial strategies to improve flexible navigation and life-space mobility. Moreover, the individuals’ abilities for egocentric and allocentric spatial orientation should be integrated into the overall testing. This will help to identify dependencies between cognitive performance and task execution as well as adaptions to the training.

The analysis of the completion time showed that the use of cognitively demanding materials (floor plans) requires more time than the other exercises. On the other hand, this task seems to be highly motivating, supported by the fact that one participant even took the floor plan with her to continue practicing (NH-C). The overall observation of the training session suggests that in general more time should be planned for these exercises to enable the participants to complete them in a calm and concentrated manner. The comparison of the initial implementation and the progression shows no significant differences comparing the initial implementation and the progression of the performance score (Fig. 2). We therefore conclude that the motor and cognitive demand was increased adequately to the individuals’ abilities over time. In summary, the results showed that the novel spatial orientation tasks are feasible for the target group: after the slight modifications mentioned above, they can be transferred into multicomponent cognitive-motor training as reported by Bischoff et al. (2020).

Regarding practical aspects within the setting, the implementation of the spatial orientation cognitive-motor training was accepted within the target group and the nursing staff. The adherence in NH‑B and NH‑C improved after the modifications due to the experiences of the first implementation in NH‑A. Key factors were the support of the nursing staff, who reminded the participants of the training and accompanied them (NH-B), and the implementation as a group training. The importance of social interaction of training conducted in groups became particularly apparent when the training sessions in NH‑C were restricted to a maximum group size of three persons: one of the participants dropped out, others reported less motivation. The implementation of the training was affected both in NH‑B, but much more in NH‑C due to COVID-19-related restrictions, which potentially biases the reasons for drop-outs. NH‑B received the training after a period of in-room isolation of their residents, several of the participants reported that they enjoyed the purposeful engagement with their environment very much. To determine the effectiveness of the training beyond our initial results and the subjective appreciation of the program, a randomized controlled trial will be conducted.

### Satisfaction with life and mobility

Next to the feasibility, the possible effects on satisfaction with life and motor performance were addressed in the evaluation. Statistical comparisons of pre- and post-training data showed no significant difference for satisfaction with life (SWLS). However, a positive trend can be discerned (*p* = 0.08 for SWLS in NH-B). This finding seems consistent with intervention effects on SWLS found in nursing home residents that were mobile (Bischoff et al., 2020) and unable to walk (Cordes, Zwingmann, Rudisch, Voelcker-Rehage, & Wollesen, 2021).

There was no statistically significant change in the SPPB score between baseline and post-test, but an increase in average for NH‑A (+0.1 point) and NH‑B (+1.6 points). Without a training intervention, nursing home residents showed decreases of 2.8% in mobility (SPPB) per month (Masciocchi, Maltais, Rolland, Vellas, & de Souto Barreto, 2019). As this decline was not observed within our data, this might support the motor benefits of our spatial orientation cognitive-motor training. Furthermore, Perera, Nace, Resnick, and Greenspan, (2018) declared an increase of 0.5 points in SPPB as relevant. Since the preservation of resources is a valuable goal for interventions in older age, this trend can be considered positive and our hypothesis can be accepted. As a consequence, it can be assumed that the training integrating the novel tasks might be as effective as previous cognitive-motor training interventions in nursing home settings (Bischoff et al., 2020; Rezola-Pardo et al., 2019). They did not find significant improvement as well, but the scores of the SPPB increased comparably after the interventions (mean 0.7 and 1.17 points). Nevertheless, to prove this hypothesis and to gain information on generalizability, future randomized controlled trials with active control groups (e.g., as addressed in the study protocol by Wollesen et al., 2020b) need to be conducted. Besides, future studies should also prove the impact of these exercises on life-space mobility, which could not be controlled within this approach. A study showed that the life space of nursing home residents is predominantly limited to the patient’s room and the dining room (Jansen et al., 2017). To counteract this, the next step is to examine in a randomized controlled trial (RCT) whether a spatial orientation cognitive-motor training has positive effects on life-space mobility and spatial orientation. This study examined the feasibility of the novel spatial orientation exercises that are required to implement the spatial orientation cognitive-motor training to nursing home residents.

## Limitations

Next to the strengths of this study, there are some limitations. This feasibility study was planned according to the guidelines of the reference framework (El-Kotob & Giangregorio, 2018; Lancaster & Thabane, 2019) as a controlled trial including pre- and post-testing. However, it appears critical that the planned control groups could not be implemented. Since it was mainly implemented in 2020, the study design had to be adapted continuously, due to COVID-19-related restrictions on the access of external persons to the facility and group size. For NH‑B and NH‑C, randomization and implementation of a control group were not possible. In addition, in NH‑B only the SWLS and the SPPB could be assessed and no post-tests were applicable in NH‑C. In NH‑C, one participant took the plan with her after the initial implementation of the exercise “Floor plan bingo”. Since no post-tests were conducted, the results were not influenced. In the following RCT (cf. study protocol, Wollesen et al., 2020b), an intention-to-treat analysis should be performed to limit influences caused by target-group-specific behavior variations.

Staff support for nursing home residents varies intra-structurally from facility to facility. Since residents in NH‑B were regularly reminded to participate in training, this may has influenced the feasibility results. However, an assessment of the participants’ perceived exertion would have been a valuable addition, e.g., using a well-established tool such as the Borg scale (Borg, 1982).

## Conclusion

The structured analysis of the feasibility of spatial orientation exercises for nursing home residents revealed that the support of the nursing staff is very important to promote regular participation in the training. Furthermore, it resulted in valuable modifications regarding instructions, complexity, and materials.

The novel spatial orientation training is feasible for residents in nursing home facilities and can be recommended as part of a multidisciplinary treatment approach. Future research should test this spatial orientation training in a larger sample for its effectiveness in terms of spatial orientation abilities and life-space mobility. Therefore, this feasibility study is followed by a randomized controlled trial as part of the PROfit project, which also adds follow-up measurements including detraining phases to the study design.

For some participants, intentional promotion of their spatial orientation ability was a new experience. To prevent overwhelming demands and resulting training disengagement we summarized the examined feasibility components as well as the first analyses of effectiveness. Therefore, our recommendations for the future implementation of spatial orientation cognitive-motor training in nursing home residents are (a) instructions of demanding spatial tasks should be accompanied by an example task, especially if materials addressing the allocentric reference frame are used (like floor plans), (b) trainers should be encouraged to adjust task complexity and materials on an individual basis, (c) acceptance of the training should be promoted among nursing staff, and (d) surroundings with as little disturbance as possible should be selected for training.

## Declarations

This study was performed in accordance with the latest version of the Declaration of Helsinki (2013). This trial was granted permission by the local ethics committee faculty of psychology and human movement science, University of Hamburg (No. 2019_248 and 2020_310). Informed consent was obtained from all individual participants included in the study.
